# Preeclampsia and its relationship to pathological brain aging

**DOI:** 10.3389/fphys.2022.979547

**Published:** 2022-10-17

**Authors:** Abigail A. Testo, Carole McBride, Ira M. Bernstein, Julie A. Dumas

**Affiliations:** ^1^ Neuroscience Graduate Program, University of Vermont, Burlington, VT, United States; ^2^ Department of Obstetrics, Gynecology and Reproductive Sciences, University of Vermont, Burlington, VT, United States; ^3^ Department of Psychiatry Larner College of Medicine, University of Vermont, Burlington, VT, United States

**Keywords:** brain aging (normal), dementia, Alzheimer’s disease, vascular dementia, preeclampsia

## Abstract

The development of preeclampsia during pregnancy may have long-term effects on brain aging in women. Associations between preeclampsia and vascular dementia have been established, however the connection between preeclampsia and Alzheimer’s disease has not been as thoroughly explored. Both preeclampsia and Alzheimer’s disease have been associated with misfolded amyloid beta proteins and inflammation; due to these similarities, in this minireview, we examined the potential links between a history of preeclampsia and the development of dementia. We also discussed how hypertensive disorders of pregnancy may relate to both normal brain aging and dementia to highlight the need for additional research regarding the long-term cognitive effects of preeclampsia on the brain.

## Introduction

**FIGURE 1:**
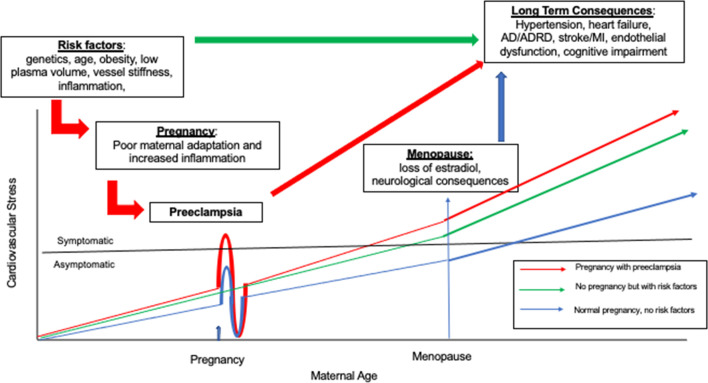
Is a model of the how women’s reproductive life events unmask different cardiovascular, metabolic, brain risk trajectories illustrating the relationship between cardiovascular stress and maternal age. The first clinical event identifying an individual’s increased risk profile is the development of preeclampsia in pregnancy. Then as aging continues, these same risk factors influence long term health consequences such as heart disease and Alzheimer’s Disease (AD). Three patterns are represented. There is variability round each of these patterns and not all women are symptomatic for cardiovascular stress. The red line illustrates women who have underlying cardiovascular and metabolic risk factors who then have a pregnancy. These women are highly likely to experience preterm preeclampsia during pregnancy with clinically significant symptoms of preeclampsia such as hypertension and proteinuria. After a first pregnancy there is some short-term protection for subsequent pregnancies represented by the undershoot, but this protection is time limited and the risks for poor health outcomes increase as aging continues. The green line represents a woman with cardiac and metabolic risk factors who does not have a pregnancy but still is at increased risk for poor cardiac, metabolic, and brain outcomes. The blue line represents a woman with no risk factors and a normal pregnancy. As each woman ages, the long term consequences of events that occur earlier in life become increasingly relevant for brain health.

Pregnancy is often referred to as a natural stress test, owing to its potential to expose previously undetected underlying conditions within the cardiovascular system (Osol and Bernstein, 2014). One condition that may develop when the body is unable to meet the hemodynamic demands associated with pregnancy is preeclampsia, which affects roughly 1 out of every 25 pregnancies in the United States according to the American College of Obstetricians and Gynecologists’ Task Force on Hypertension in Pregnancy (Hypertension in Pregnancy, 2013). Preeclampsia is a condition characterized by the combination of hypertension during pregnancy coupled with damage to other organ systems, most commonly the liver or kidneys, during the second half of pregnancy. Risks of preeclampsia include placental abruption, eclampsia, and HELLP (Hemolysis, Elevated Liver enzymes, and Low Platelets) Syndrome, all of which can be life threatening. Beyond the more immediate risks associated with preeclampsia, the condition may signal that an individual’s cognitive health may be at risk later in life as it may expose underlying risk factors which contribute to long-term consequences including hypertension and stroke. While the field has not yet reached a consensus regarding preeclampsia’s long-term risks as they relate to brain aging, evidence has begun to immerge that establishes an interaction between the two. Figure 1 is a model of the effects of reproductive history on brain aging in women. We propose that the underlying cardiovascular and metabolic processes contribute to preeclampsia in women during pregnancy but may also exist in women who do not have a pregnancy. These risk factors and their progression to preeclampsia contribute to the development of pathologic brain processes as a woman ages. We use this minireview to evaluate the proposal that events observed during pregnancy have long-term consequences on the brain in women.

Women with a history of hypertensive disorders of pregnancy report increased subjective cognitive complaints compared to women with normotensive pregnancy postpartum (Aukes et al., 2007; Postma et al., 2013; Mielke et al., 2016). Furthermore, studies using objective measures of cognition similarly demonstrated that women with a history of hypertension in pregnancy approximately 15 years prior performed worse on tests of verbal memory and learning than women with normotensive pregnancies (Mielke et al., 2016; Adank et al., 2021). Differences in structural neuroanatomy have also been detected where women with a history of preeclampsia have reduced cortical grey matter, decreased temporal lobe white matter microstructure, increased temporal lobe white matter lesion volume, and altered microstructural integrity compared to women with no history of any hypertension in pregnancy (Mielke et al., 2016; Siepmann et al., 2017). In addition, white matter microstructural changes increased with time since pregnancy and demonstrated a trajectory of decline in women with a history of preeclampsia but not those who were normotensive during pregnancy (Siepmann et al., 2017). Taken together these studies provide evidence to support investigations into how preeclampsia may affect afflicted individuals even decades after diagnosis.

Women who developed preeclampsia are also at increased risk for both stroke and vascular dementia (VaD), the second most common form of dementia (Zambrano and Miller, 2019). VaD, however, may not be the only form of dementia in which preeclampsia may play a role as previous studies have also found that a history of preeclampsia may also increase an individual’s likelihood of developing Alzheimer’s disease (AD) (Theilen et al., 2016). AD is characterized by the deposition and accumulation of amyloid-beta peptides and the presence of neurofibrillary tangles (Yue et al., 2005; Au et al., 2016) as well as by the increased presence of inflammation within the brain. One potential link between preeclampsia and AD is evidence of toxic protein misfolding in preeclampsia and amyloid precursor protein is one of the proteins found to aggregate in both the plasma and placenta (Buhimschi et al., 2014).

By examining the potential link between preeclampsia and AD and the established link between preeclampsia and VaD, it may be possible to investigate preeclampsia’s long-term association with brain aging in women, however, the existing literature dedicated to such an investigation is far from complete. A previous review by Erez et al. (Erez et al., 2022) detailed how preeclampsia is understood to be a vascular condition exposed by pregnancy and may cause a subset of women to be at increased risk for developing VaD later in life. However, the main focus of the review was on the conceptual evolution of preeclampsia as a disorder around pregnancy not the effects of preeclampsia later in life. In this mini review we will discuss potential links between a history of preeclampsia and the development of dementia in older ages, as well as how, or whether, hypertensive disorders of pregnancy distinguished normal brain aging from dementia. We then discuss findings related to potential underlying mechanisms of any such links. Lastly, we discuss possible interventions to improve long-term health outcomes following preeclampsia diagnosis as well as their implications for normal and pathological brain aging.

## Cognitive impairment in late life

Pregnancy often occurs decades before the development of dementia, making it difficult to study how complications related to pregnancy affect the long-term cognitive health of the mother. A small number of studies, however, have begun to investigate the potential links between a history of preeclampsia and cognitive health. A review by Elharram et al. (Elharram et al., 2018) found no clear evidence of impairment on standard neurocognitive tests in young women with a median of 6 years post pregnancy. However, it is likely that the relationship between preeclampsia and cognitive decline takes longer to develop and appears in older ages. Fields et al. (Fields et al., 2017) investigated whether women were at increased risk of cognitive decline approximately 35–45 years after the development of preeclampsia. They found that while raw cognitive and mood scores did not significantly differ between the preeclampsia and normotensive pregnancy groups, a trend towards mild cognitive impairment or dementia occurred more frequently among women with a history of preeclampsia. This trend also affected more cognitive domains within the preeclampsia group including executive dysfunction and verbal list learning impairment (Fields et al., 2017). It may be the case that cognitive impairment later in life become becomes increasingly likely when an individual has both a history of preeclampsia and other vascular conditions. One study found that women in midlife with a history of preeclampsia and maternal vascular malperfusion (MVM), a complication of pregnancy in which decidual vasculopathy, villous infarction, accelerated villous maturation, perivillous fibrin deposition, or intervillous fibrin deposition are found in the placenta, had worse information processing speed compared to both women with a history of preeclampsia, but no history of MVM and women who had no history of either disorder (Shaaban et al., 2021).

Additional studies have found stronger associations between a history of preeclampsia and dementia in later life. Those with a history of preeclampsia may be as much as three times more likely to develop VaD compared to those who did not have such a history even after adjusting for diabetes, hypertension, and cardiovascular disease, with the association being greater for late onset dementia (age 65≥) than early onset dementia (age<65) (Basit et al., 2018). An increased risk of VaD was reported in women with a history of preeclampsia and in women with previous pregnancy-induced hypertension, spontaneous preterm labor and birth, or preterm premature rupture of membranes with the association remaining after adjusting for cardiovascular disease and socio-demographic factors (Andolf et al., 2020). While associations between preeclampsia and VaD have been consistently shown, less is known about the relationship between preeclampsia and AD.

Investigations of preeclampsia’s potential link to AD have yielded some positive results. Modest associations between preeclampsia and AD have been reported (Basit et al., 2018), as has an increased risk of mortality from AD in women with a history of preeclampsia (Theilen et al., 2016). Additionally functional changes among women with a history of preeclampsia including memory loss, attention deficit and motor speed impairment are similar to those seen in AD pathology (Ijomone et al., 2018).

Findings linking preeclampsia to dementia are not yet conclusive, however. A study by Andolf et al. (Andolf et al., 2017) found no increased risks for VaD or dementia after any hypertensive disorders in pregnancy, however, the researchers themselves noted the wide confidence interval within their dataset and risk of misclassification of participants. Further studies are needed based on concerns due to the pathophysiology of preeclampsia, the presence of brain lesions, as well as the increased risk for cardiovascular disease (Andolf et al., 2017). One other study similarly found no increased risk of dementia associated with pregnancy hypertensive disease (Nelander et al., 2016). Despite the results of these two studies, the studies described above have demonstrated associations between a history of preeclampsia and cognitive impairments and dementia later in life, thus additional investigations to better understand these associations are warranted.

## Neuroinflammation

Inflammation resulting from the secretion of pro-inflammatory cytokines is commonly recognized in cases of illness and infection, such as the development of fever; however, subclinical, chronic low-grade inflammation may be a contributing factor to brain aging (Minciullo et al., 2016). Inflammation may explain why otherwise healthy adults experience cognitive deficits; specifically, circulating inflammatory biomarkers have been linked to declines in cognitive functioning and worsening of structural and metabolic characteristics, including volumetric reductions and amyloid deposition (Rosano et al., 2012). Inflammation is a common symptom of traumatic brain injury, diabetes, hypertension, and obesity, all of which increase the risk of developing AD. AD increased characterized presence of inflammation within the brain, as well as the deposition and accumulation of amyloid-beta peptides and the presence of neurofibrillary tangles (Yue et al., 2005; Au et al., 2016). Chronic inflammation is believed to play a role in the onset and progression of AD and links between inflammation and several age-related diseases continue to emerge (Newcombe et al., 2018). Recent studies have begun to link neuroinflammation and injury to the blood brain barrier to the development of preeclampsia. Women who developed preeclampsia had increases in the pro-inflammatory cytokines interleukin-6 (IL-6) and interleukin-8 (IL-8) in their cerebral spinal fluid compared to women who had normotensive pregnancies; furthermore, women with eclampsia showed elevated levels of IL-6, IL-8, and tumor necrosis factor alpha (TNF-α) in their cerebral spinal fluid (Bergman et al., 2021). Patients with preeclampsia also showed elevated levels of the proteins transforming growth factor-β (TGF-β), vascular endothelial growth factor A (VEGFA), and angiotensinogen in their cerebral spinal fluid (Ciampa et al., 2018). The inflammatory response of the immune system in cases of preeclampsia may be in part a result of interactions between proteins and macrophages. A high-affinity binding protein for the pleiotropic inflammatory cytokine macrophage migration inhibitory factor called cluster of differentiation 74 was downregulated in a mouse model of preeclampsia (Przybyl et al., 2016). This led to altered macrophage activation and an increase in the secretion of TNF-α, chemokine ligand 5, and monocyte chemotactic protein-1 (Przybyl et al., 2016). Not all studies have found such conclusive results, such as Burwick et al. (Burwick et al., 2019) which did not find evidence of blood brain barrier damage or inflammation in case of preeclampsia; however, a majority of studies indicate a pro-inflammatory signature of preeclampsia similar to that of AD.

## Misfolded proteins

The deposition of misfolded protein aggregates in the brain is a feature of many neurodegenerative diseases including AD, which is characterized by the deposition and accumulation of amyloid-beta peptides and the presence of neurofibrillary tangles (Yue et al., 2005). For this reason, the presence of misfolded proteins is an important area of investigation in AD research. Recent research has identified preeclampsia as a proteinopathy disease due to detectable misfolded proteins being found in the urine (Buhimschi et al., 2014) and serum of preeclampsia patients (Cheng et al., 2021). Proteoforms of ceruloplasmin, immunoglobulin free light chains, SERPINA1, albumin, interferon-inducible protein 6–16, and β-amyloid were all found in the urine of women with preeclampsia when a Congo red dot test was positive (Buhimschi et al., 2014). Further evidence to suggest that preeclampsia, like AD, is a disease of misfolded proteins may be found in investigations of the placentas of women with preeclampsia. Amyloid precursor protein-processing enzymes α-secretase ADAM10, the β-secretases BACE1 and BACE2, and the γ-secretase presenilin-1 found in the placenta have all been found to be up-regulated in cases of preeclampsia (Buhimschi et al., 2014); as β-amyloid aggregates, their presence establishes yet another link between preeclampsia and AD.

Among disorders that have protein misfolding as a part of their pathology, preeclampsia is unique because many proteinopathy diseases are age related. One potential explanation for the presence of misfolded proteins in cases of preeclampsia is a disruption in the chaperone functioning of pregnancy zone protein. Pregnancy zone protein efficiently inhibits *in vitro* aggregation of misfolded proteins, specifically β-amyloid. In severe cases of preeclampsia, pregnancy zone protein-positive extravillous trophoblasts found in the placenta were adjacent to extracellular plaques containing β-amyloid; however, concentrations of pregnancy zone protein within the extracellular plaques that contained β-amyloid were low (Cater et al., 2019). Another potential regulator of misfolded proteins is glycosylated plasminogen activator inhibitor type-2 (PAI-2), which is upregulated in pregnancy and in response to inflammation. Research has shown that glycosylated PAI-2 inhibits the aggregation of β-amyloid peptides and is implicated in cases of preeclampsia and AD (Cater et al., 2022). Anti-aging protein α-klotho also differs in concentration by pregnancy outcome and studies have shown maternal plasma levels of α-klotho were elevated in preeclampsia (Loichinger et al., 2016). These studies provide evidence to support the proposal that preeclampsia, like AD, is associated with significant proteinopathy, however, further investigation is needed to determine what the underlying cause of misfolded proteins in the cases of preeclampsia.

## Cerebral biomarkers and blood-brain barrier integrity

Cerebral biomarkers for neurological injury in dementia include neurofilament light and tau found in axons, as well as neuron-specific enolase and S100B found in glial cells (Friis et al., 2022). Recent studies investigated whether these cerebral biomarkers for neurological injury were present in cases of preeclampsia and the findings were inconsistent. A 2021 study found increased neurofilament light chain concentrations in both plasma and cerebral spinal fluid in cases of preeclampsia, indicating neuroaxonal injury (Andersson et al., 2021). Additionally, the same study found cerebral spinal fluid concentrations of neuron-specific enolase and tau were decreased in preeclampsia, however, the same decreases were not seen in plasma concentrations. No difference was found in cerebral spinal fluid concentrations of S100B between women with preeclampsia and normal pregnancy, however, serum concentrations of S100B were elevated in cases of preeclampsia (Andersson et al., 2021). Other studies have similarly found plasma neurofilament light, glial fibrillary acidic, and tau to all be identified as potential predictors of cerebral complications in cases of preeclampsia (Bergman et al., 2022), with neurofilament light in particular potentially being a predictor of preeclampsia in older women (Evers et al., 2018). The existing literature is not entirely consistent, however. Friis et al. (Friis et al., 2022) reported increased plasma concentrations of neurofilament light, tau, neuron-specific enolase, and S100B in women with preeclampsia compared to women who had normative pregnancies. Conflicting findings such as those related to S100B highlight the need for additional research regarding the underlying influences of cerebral biomarkers. Cerebral biomarker levels may be influenced by gestational age at preeclampsia onset. Neurofilament light chain was significantly increased in women who developed preeclampsia in gestational week 33 and 37, while tau concentrations were increased when preeclampsia developed beyond gestational week 37 (Bergman et al., 2018).

Cerebral biomarkers were associated with blood-brain barrier integrity. Higher concentrations of neurofilament light chain were associated with decreased transendothelial electrical resistance, indicating damage to the blood-brain barrier (Friis et al., 2022). Additional evidence suggested the blood-brain barrier may be injured in both preeclampsia and eclampsia. Women who developed either preeclampsia or eclampsia showed an increased cerebral spinal fluid to albumin ratio, indicating injury to the blood-brain barrier, compared to women with normotensive pregnancies (Bergman et al., 2021). These neuroaxonal injuries and damage to blood-brain barrier that develop with preeclampsia may increase the risk for dementia that develops decades after the afflicted pregnancy.

## Brain structural changes

Preeclampsia may result in long term structural changes to the brain which may increase the risk for dementia. Women with severe preeclampsia had cerebral white matter lesions at the time of delivery, the majority of which were found in the frontal lobes; these white matter lesions were still seen in a majority of cases 6 months postpartum with slightly less than half persisting up to one-year postpartum (Soma-Pillay et al., 2017). It is unclear what the underlying cause of these lesions was and the recovery of some lesions points to less permanent pregnancy-related brain pathology. However, some lesions persisted at least 1 year postpartum. In addition to white matter lesions, volumetric changes were observed in preeclampsia, even after controlling for total intercranial volume. Observations of smaller brain volumes in women with a history of preeclampsia compared to women with a history of normative pregnancies, as well as, increased presence of white matter lesions were found decades after the affected pregnancy, suggesting that brain atrophy may have occurred (Mielke et al., 2016). Brain atrophy and the presence of white matter lesions were combined to form a composite measure called brain injury and higher brain injury measures resulted in slower processing speeds (Mielke et al., 2016). These volumetric changes may be related to alterations to the permeability of the blood brain barrier. Blood brain barrier leakage in both white and grey matter was greater in women with a history of preeclampsia than women who had a normative pregnancy (Canjels et al., 2022). These results demonstrated that injury to the brain in cases of preeclampsia may have lasting effects that persist as women age.

## Genetics

Genetic components may play a role in the development of both preeclampsia and dementia. Evidence of common genetic factors in preeclampsia and AD include STOX1A expression which is associated with preeclampsia and is correlated with late onset AD (van Abel et al., 2012). STOX1A has been found to downregulate the expression of the CNTNAP2 gene in the hippocampus that was associated with late onset AD (van Abel et al., 2012) and late onset AD severity (van Dijk et al., 2010). Previous studies have also demonstrated that pregnancy affected the cytotrophoblast epigenome and if the required changes are not able to be made to the epigenome, preeclampsia may be the ultimate result (Zhang et al., 2021). Additionally, in cases of severe preeclampsia, H3K27 acetylation was increased globally and at genes that were normally downregulated at term but upregulated in preeclampsia, which may be associated with premature aging (Zhang et al., 2021).

Results examining a potential underlying genetic connection between preeclampsia and pathological brain aging are far from conclusive as not all previous studies have consistently found correlations between brain aging and genes believed to be involved in preeclampsia. Lysosomal glucosidase beta acid (GBA) deficiency is common in Lewy body dementia and while increased lysosomal GBA is not currently linked to any human disease, GBA RNA expression is upregulated in preeclamptic placentae. Investigations into how lysosomal GBA relates to a common biomarker for preeclampsia, sFLT1, however, found no correlation between lysosomal GBA and FLT1 (Jebbink et al., 2015). Additionally, some studies have found more inconclusive results regarding the genetic susceptibility to preeclampsia; such as a study by Freed et al. (Freed et al., 2005) that found no difference in trinucleotide repeats (CAG) in normotensive and severe pre-eclampsia. These results demonstrated that while there is likely genetic susceptibility for both preeclampsia and dementia, more research is necessary before a conclusion may be drawn about the connections between them.

## Discussion

The studies reviewed above support the proposal that a history of preeclampsia increases an individual’s risk for developing cognitive impairments later in life, including both VaD and AD (Basit et al., 2018); however, the mechanism by which this risk is increased warrants further investigation as some studies had conflicting findings. Evidence in support of a link between preeclampsia and pathological brain aging is strengthened by findings such as the presence of toxic protein misfolding in both AD and preeclampsia, as well as, the presence of amyloid precursor protein and increased proinflammatory cytokines in cases of preeclampsia and AD (Buhimschi et al., 2014; Minciullo et al., 2016). The lack of consistent findings regarding underlying genetic factors indicates that a clear genetic association is not currently known and further study is needed.

A barrier to conducting these studies is the timeline, with decades between the afflicted pregnancy and the first signs of dementia, thus it is difficult to design studies investigating the relationship between preeclampsia and dementia. While this long-time frame causes difficulties for researchers, it can provide hope for patients. Certain pre-pregnancy specific phenotypes are predictive of preeclampsia including increased android body fat, increased supine pulse, increased supine blood pressure, higher cardiac output, increased beta adrenergic responsiveness, increased popliteal pulse wave velocity, reduced renal resistance, increased C-reactive protein (CRP), elevated soluble alpha amyloid precursor protein α (sAPPα), and an exaggerated response to volume loading compared to women who did not develop preterm preeclampsia (Bernstein et al., 2017). As many of these risk factors for preeclampsia overlap with the risk factors for developing dementia, most notably VaD due to the cardiovascular nature of both preeclampsia and VaD, it may be possible for an individual to take steps to mitigate their risk for dementia in the long term once their preeclampsia is resolved. This would require behavioral changes, including controlling high blood pressure, maintaining healthy cholesterol levels, not smoking, maintaining a healthy body weight, and being physically active (Tariq and Barber, 2018). While behavioral changes such as these are often difficult to achieve, the time frame between the affected preeclampsia and the development of dementia does allow ample time to act. Additionally, discovery of a connection between preeclampsia and dementia itself may help better identify and target the pathology that underlies dementia. Women are diagnosed with AD at a rate of two to one when compared to men, a disparity not accounted by the increased longevity of women alone (Au et al., 2016). Furthermore, additional research is required to better characterize the subgroup among preeclampsia patients that puts some women at risk for subsequent cognitive impairment, as currently the aforementioned subpopulation is obscured due to the heterogeneity of the preeclamptic phenotypes.
