# Corrigendum: Preeclampsia and its relationship to pathological brain aging

**DOI:** 10.3389/fphys.2022.1096042

**Published:** 2022-12-12

**Authors:** Abigail Testo, Carole McBride, Ira M. Bernstein, Julie Dumas

**Affiliations:** ^1^ Neuroscience Graduate Program, University of Vermont, Burlington, VT, United States; ^2^ Department of Obstetrics, Gynecology and Reproductive Sciences, University of Vermont, Burlington, VT, United States; ^3^ Department of Psychiatry Larner College of Medicine, University of Vermont, Burlington, VT, United States

**Keywords:** brain aging (normal), dementia, Alzheimer’s disease, vascular dementia, preeclampsia

In the published article, there was an error. We stated that recent research had identified preeclampsia as a proteinopathy disease because misfolded proteins were found in urine and serum. We cited Cheng et al., 2021 with that statement. However, Cheng et al., 2021 found misfolded proteins in serum and did not study urine. We did not include Buhimschi et al., 2014, who made the finding in urine in the specified place in this paragraph. This statement, as mistakenly written, misrepresents the work of Cheng et al., 2021 and does not properly cite Buhimschi et al., 2014. As such, a correction has been made to **Misfolded Proteins**, paragraph 1 of the paper. The sentence previously stated:

“Recent research has identified preeclampsia as a proteinopathy disease due to detectable misfolded proteins being found in the urine and serum of preeclampsia patients (Cheng et al., 2021).”

The corrected sentence appears below:

“Recent research has identified preeclampsia as a proteinopathy disease due to detectable misfolded proteins being found in the urine (Buhimschi et al., 2014) and serum of preeclampsia patients (Cheng et al., 2021).”

The authors apologize for this error and state that this does not change the scientific conclusions of the article in any way. The original article has been updated.

